# Supplementary material for: Genetic Analysis for Resistance to Sclerotinia Stem Rot, Yield and Its Component Traits in Indian Mustard [Brassica juncea (L.) Czern & Coss.]
Source: Plants (Basel). 2022 Feb 28;11(5):671. doi: 10.3390/plants11050671 (PMC8912491; doi:10.3390/plants11050671)
Supplement: Supplementary file 1 [file plants-11-00671-s001.zip › plants-1564758-si.pdf]

Supplementary Table S1: Weekly averaged meteorological data during crop season of 2019-20 at CCS Haryana Agricultural University, Hisar

Standard Meteorological Weeks	Dates	Temperature (° C)		Relative humidity (%)		Total Rainfall (mm)
		Maximum	Minimum	Morning	Evening	
42	15 October - 21 October	34.1	18.4	79	34	0.0
43	22 October - 28 October	31.9	14.9	79	31	0.0
44	29 October - 04 November	30.7	16.2	90	40	0.0
45	05 November - 11 November	28.4	12.7	85	36	0.3
46	12 November - 18 November	26.8	12.7	86	41	0.0
47	19 November - 25 November	26.7	10.9	88	42	0.0
48	26 November - 02 December	22.6	12.1	92	62	12.0
49	03 December - 09 December	23.1	6.0	88	47	0.0
50	10 December - 16 December	19.2	8.3	95	73	4.5
51	17 December - 23 December	13.7	6.1	99	81	0.0
52	24 December - 31 December	11.9	2.6	97	75	0.0
1	01 January - 07 January	17.3	5.7	96	60	Trace
2	08 January - 14 January	17.7	5.7	96	64	3.2
3*	15 January - 21 January	13.4	4.7	100	82	0.0
4*	22 January - 28 January	19.2	5.0	96	56	7.2
5*	29 January - 4 February	18.8	3.9	98	61	0.0
6*	05 February - 11 February	20.1	2.8	92	46	0.0
7	12 February - 18 February	24.7	4.8	93	36	0.0
8	19 February - 25 February	23.8	10.5	89	61	10.9
9	26 February - 04 Mar	26.0	12.1	95	55	0.0
10	05 March - 11 March	23.1	11.7	90	66	61.8
11	12 March - 18 March	23.8	9.8	94	53	11.6
12	19 March - 25 March	29.2	14.4	90	52	1.5
13	26 March - 01 April	27.6	15.3	92	54	20.1

*Weeks where *S. sclerotiorum* inoculation and disease assessment was done.
